# Temporal Relationship between Diet-Induced Steatosis and Onset of Insulin/Leptin Resistance in Male Wistar Rats

**DOI:** 10.1371/journal.pone.0117008

**Published:** 2015-02-06

**Authors:** Li Zhang, Haiyan Song, Yingli Ge, Guang Ji, Zemin Yao

**Affiliations:** 1 Institute of Digestive Disease, Longhua Hospital, Shanghai University of Traditional Chinese Medicine, Shanghai 200032, China; 2 E-institute of Shanghai Municipal Education Commission, Shanghai University of Traditional Chinese Medicine, Shanghai 200032, China; 3 Department of Biochemistry, Microbiology & Immunology, Ottawa Institute of Systems Biology, University of Ottawa, Ottawa, Canada; Hosptial Infantil Universitario Niño Jesús, CIBEROBN, SPAIN

## Abstract

Rats fed with high-fat-high-sucrose (HFHS) diet are known to manifest metabolic syndrome including hyperinsulinemia, hyperleptinemia, hyperglycemia, diabetic dyslipidemia, and hepatic steatosis. The aim of the current study is to determine the temporal relationships between the development of hepatic steatosis and the onset of insulin and leptin resistance in hypothalamus and liver in male Wistar rats (six weeks of age) fed chow or HFHS diet for up to 8 weeks. Fasting plasma glucose, lipids/lipoproteins, insulin and leptin levels were quantified, histopathologic score of hepatic steatosis and inflammation were assessed, and the responses of common checkpoints of insulin and leptin signalling responsible for lipogenesis and gluconeogenesis were analyzed. In addition, acute insulin or leptin administration was performed at different stages of HFHS dieting to determine the responsiveness of the respective signalling pathways. Hyperinsulinemia, hyperglycemia, dyslipidemia, and increased homeostasis model assessment of basal insulin resistance occurred 1-week after HFHS dieting, coinciding with upregulation of suppressor of cytokine signalling 3 in both hypothalamus and liver. However, hepatosteatosis, accompanied with increased expression of sterol regulatory element binding protein 1c and phosphoenolpyruvate carboxykinase, did not manifest until 4- to 8-week after HFHS dieting. Lowered insulin sensitivity (shown by decreased insulin receptor substrate 1 and protein kinase B phosphorylation) occurred approximately 2 weeks prior to leptin resistance (shown by impaired signal transducer and activator of transcription 3 activation) in both the liver and hypothalamus. Acute insulin/leptin administration also demonstrated the impaired insulin or leptin signalling transduction. These data suggest that lowered insulin sensitivity and leptin resistance occurred at least 2–3 weeks earlier than the manifestation of hepatosteatosis in rats fed HFHS diet.

## Introduction

Nonalcoholic fatty liver disease (NAFLD) has become a common form of chronic liver disease worldwide, affecting one third of populations [1]. Accumulation of lipid, mainly triglyceride (TG), is considered the main feature of NAFLD, with steatosis being the pathological status in clinics. Pathology of NAFLD encompasses a spectrum of abnormalities, ranging from simple steatosis to nonalcoholic steatohepatitis, fibrosis, and eventual cirrhosis [2,3]. In addition to these hepatic abnormalities, NAFLD is also a contributing factor of metabolic syndrome, a cluster of lipid and lipoprotein disorders closely associated with type 2 diabetes and premature cardiovascular disease [4].

Although the mechanisms underlying the pathogenesis of NAFLD are not fully understood, available data from human and animal studies have indicated a link between insulin and/or leptin resistance and NAFLD [5–10]. Insulin signalling sensitizers (e.g. pioglitazone and metformin) that commonly used in treating diabetes have proven clinically beneficial in improving biochemical indices in NAFLD patients [11]. Similarly, improving insulin sensitivity can also lead to decreased hepatic steatosis in animals [12]. Moreover, strategies that improve both insulin and leptin sensitivity have demonstrated promising outcomes in attenuating NAFLD [13].

The adipose-derived hormone leptin is known to act on hypothalamus to reduce food intake and increase energy expenditure. Studies with animals fed with a high-fat diet invariably show hyperleptinemia and hyperinsulinemia under diet-induced-obesity (DIO) conditions [14]. Hyperleptinemia exerts no anorexic effect in the DIO animals, suggesting a state of leptin resistance in hypothalamus [15]. Leptin resistance in hypothalamus is associated with up-regulation of suppressor of cytokine signalling 3 (SOCS3) and subsequent down-regulation of signal transducer and activator of transcription 3 (STAT3) activation [16,17]. Activation of STAT3 is achieved by phosphorylation catalyzed by Janus kinase 2 (JAK2), a tyrosine kinase associated with OBR [18], whereas SOCS3 participates as a suppressor in the negative feedback loop attenuating STAT3 mediated signaling [19]. SOCS3 attenuates leptin signaling through several mechanisms, including direct binding to OBR and/or tyrosine-phosphorylated JAK2 and thus inactivating JAK2 [20].

Recent experimental data have suggested that leptin can act directly on the liver, influencing the insulin signalling pathway in addition to the leptin signalling pathway, and therefore on hepatic lipid and lipoprotein metabolism [21,22]. Functional OBR, both the long and the short forms (designated OBR_L_ and OBR_S_, respectively), were expressed in the liver [23]. Gain or loss of hepatic leptin action in mice has a profound impact on the phosphatidylinositol 3 kinase (PI3K) activity and hepatic steatosis (i.e. TG content) [24,25]. Thus, leptin may directly exerts an effect on lipid and glucose metabolism in the liver through the PI3K pathway.

The common PI3K pathway that is shared by insulin and leptin signalling features a close cross-talk between the two hormonal regulations in lipid and glucose metabolism in both central and peripheral tissues. In central, insulin has been shown to act on the same key areas in the brain as leptin does [26]. Intracerebroventricular infusion of insulin in mice affects both food intake and lipid metabolism in peripheral tissues, whereas brain-specific disruption of the insulin receptor gene in mice resulted in disorders similar to that observed in leptin-deficient ob/ob mice [27]. In the liver, inactivation of hepatic SOCS3 resulted in increased insulin sensitivity and lipogenesis [28], whereas expression of recombinant STAT3 in the liver markedly attenuated hyperglycemia and hyperinsulinemia in diabetic mice [29]. Liver-specific inactivation of the insulin receptor in mice resulted in dyslipidemia and increase risk of atherosclerosis [30].

The present study aimed to determine changes of the insulin/leptin signalling molecules during the development of hepatosteatosis. We employed the HFHS diet-induced hyperinsulinemia, hyperleptinemia, and diabetes rat model to delineate a temporal relationship between the development of NAFLD and the onset of insulin/leptin resistance. The data indicate that the lowered insulin sensitivity occurred earlier than leptin resistance in both liver and hypothalamus. Uncontrolled hepatic glucose production and upregulation of lipogenesis were detected at late stages of HFHS dieting, which was associated with pathological manifestation of NAFLD.

## Materials and Methods

### Animals

Male Wistar rats (6 week old) were obtained from SLAC Animal Laboratories (Shanghai, China), and housed under a standard 12-h light-dark cycle (lights on at 7:00 AM) with access to food and water ad libitum. After approximately 1-week acclimation, rats were placed on chow or HFHS diet (SLAC Animal Laboratories) for up to 8 weeks. The energy content of chow diet is 4.15 kcal/g, and 100 g chow contains (in grams): casein, 20; starch, 66.07; soybean oil, 4; cellulose, 5; mineral mix, 3.5; vitamin mix, 1; L-cystine, 0.18; and choline bitartrate, 0.25. The energy content of the HFHS diet is 5.13 kcal/g and 100 g HFHS food contains (in grams): casein, 20; starch, 34.07; sucrose, 15; lard, 15; soybean oil, 4; cellulose, 5; mineral mix, 3.5; vitamin mix, 1; L-cystine, 0.18; choline bitartrate, 0.25; and cholesterol, 2. Rats were individually housed in a pathogen free environment, and body weight and food intake were measured weekly. For the acute insulin/leptin treatment experiment, rats fed with chow or HFHS diets were intraperitoneally injected with recombinant insulin (0.75 U/kg body weight) or recombinant rat leptin (0.6 mg/kg body weight), and liver samples were collected 30 min after the injection. The animal protocols were performed in accordance with the guidelines and approval of the Animal Experiment Ethics Committee at Shanghai University of Traditional Chinese Medicine.

### Oral Glucose Tolerance Test

Rats were fasted for 6 h after the start of the light cycle, and then orally administered with glucose (1.5 g/kg body weight). Tail-vein blood samples were collected at baseline and at indicated time intervals (15, 30, 60, 90, and 120 min) after glucose treatment. Blood glucose levels were determined with a diabetes monitoring strip (Lifescan One Touch, IN).

### Serum Biochemical Analysis

After fasting for 12 h, rats were anaesthetized with sodium pentobarbital (100 mg/kg) and sacrificed, and blood was collected from aorta abdominalis. Serum TG, total cholesterol (TC), high density lipid-cholesterol (HDL-c), low density lipid-cholesterol (LDL-c), free fatty acids (FFA), fasting blood glucose (FBG), alanine transaminase (ALT), and aspartate transaminase (AST) were analyzed using the Hitachi full-automatic system with corresponding kits (Wako, Richmond, VA, USA). Fasting insulin (FIN) and leptin were measured using the standard radio-immunity kits (Puerweiye Bioengineering Institute, Beijing, China). The homeostasis model assessment of basal insulin resistance (HOMA-IR) was calculated using formula FBG (mM) × FIN (IU/L)/22.5.

### Hepatic Histology Assessment

Liver sections were stained with Hematoxylin and eosin (HE) and Oil-Red O (neutral lipid), the procedures were performed according to previously describe methods [31]. Briefly, the liver tissues were fixed in 10% neutral buffered formalin for 24 h, dehydrated and embedded in paraffin, the sections were cut, deparaffinized and stained with HE. Snap frozen tissues were placed in optimal cutting temperature compound and then sectioned and stained with Oil-Red O buffer. Images were taken under Olympus IX71 Inverted microscope (Tokyo, Japan). Hepatic steatosis was graded based on the extent of lipid accumulation: <5% (score 0), 5–33% (score 1), >33–66% (score 2), and >66% (score 3) according to the histopathologic criteria specified previously [32].

### Measurement of Hepatic Lipid Content

Liver TG and cholesterol were quantified as described previously [33]. Briefly, liver tissue (200 mg) was homogenized in 3 ml of ethanol-acetone (1:1) mixture. The homogenate was extracted over night at 4°C, and centrifuged for 15 min at 3,000 rpm at 4°C. The organic layer was removed, TG and cholesterol were measured using commercial kits (Kangtai Bioengineering Institute, Beijing, China).

### Tissue Sampling and Western Blot Analysis

After euthanasia and blood collecting, hypothalamus and liver were removed, immediately frozen in liquid nitrogen and stored at -80°C. Frozen tissues were homogenized in Tissue Protein Extraction Reagent (Pierce Biotechnology, Inc., Rockford, USA), with the addition of protease inhibitor (Roche, Nutley, USA) and phosphatase inhibitor cocktail (Roche, Nutley, USA). Protein concentrations were determined using the bicinchoninic assay reagents and the micro-bicinchoninic assay method (Pierce Biotechnology, Inc. Rockford, USA). For Western blot analysis, 100 μg of protein were fractionated by SDS-PAGE (8–12% gradient gel), transferred onto a PVDF membrane (Bio-Rad, Hercules, CA), Membranes were blocked with 5% skim milk in Tris-buffered saline and probed with target primary and secondary antibodies (see S1 Table for detailed information of antibodies used in the present studies). The targeted proteins were detected with ECL Detection Kit (Millipore, Billerica, USA), images were taken and qualified by Gel-Pro system (Tanon Technologies). For western blot analysis, the amount of protein loaded was confirmed by the Bradford method, and equal loading was verified by staining with Ponceau S reagent (Sigma Chemical Co.) and by determining the signal of beta actin.

### Statistical Analysis

For each outcome measure, a one-way analysis of variance was performed (SPSS 18.0) for each animal group studied (n = 6–8). A significant main effect (*P* < 0.05 or *P* < 0.01) was followed up with Student-Newman-Kuel post hoc comparisons. Values are presented as means ± standard error of the mean (SE), and *P* < 0.05 denotes a statistically significant difference.

## Results

### Changes in serum metabolic parameters upon HFHS dieting

As expected, the HFHS dieting markedly induced a diverse range of metabolic abnormalities, including hypertriglyceridemia, hypercholesterolemia, hyperglycemia, hyperinsulinemia, as well as hyperleptinemia, and most of these abnormalities occurred as early as 1-week and remained throughout the entire HFHS dieting (Table 1). The hypercholesterolemia in HFHS diet-fed rats was associated with increased LDL-c and decreased HDL-c (Table 1), typical phenotypes of diabetic dyslipidemia. The hallmarks of lowered insulin sensitivity, including hyperinsulinemia, hyperglycemia, and increased HOMA-IR were also manifested 1-week after HFHS dieting, indicative of a rapid response of the animals in attenuating insulin signaling. Oral glucose tolerance test (OGTT) did not show significant difference between HFHS diet- and chow diet-fed animals at 1-week (Table 1), However, prolonged HFHS dieting (4, and 8-week) resulted in a trend of increased OGTT-AUC value as compared to that of chow diet-fed rats (0 week) (Table 1). These results suggest that HFHS dieting contributes to the compromised insulin sensitivity in these rats.

**Table 1:** Serum metabolic parameters and food intake.

Parameters	Chow diet (week)					HFHS diet (week)			
	0	1	2	4	8	1	2	4	8
TG (mM)	1.29±0.12	1.57±0.12	1.01±0.1	1.28±0.28	1.12±0.08	1.75±0.19	2.93±0.22**	2.41±0.25**	1.90±0.14**
TC (mM)	1.39±0.03	1.87±0.09	1.51±0.06	1.46±0.07	1.59±0.04	2.50±0.13**	3.31±0.13**	3.34±0.16**	2.95±0.13**
HDL-c (mM)	1.11±0.03	1.02±0.02	1.00±0.03	1.01±0.02	1.02±0.01	0.88±0.02	0.69±0.04**	0.72±0.02**	0.80±0.04**
LDL-c (mM)	0.19±0.01	0.41±0.03^§^	0.25±0.13	0.28±0.01	0.32±0.01	0.95±0.10**	1.37±0.06**	1.68±0.04^†^ **	1.60±0.08^†^ **
FFA (u/l)	1787±97.8	1750±60.5	1746±207.4	1743±57.1	1792±81.6	1766±57.2	1925±77.8	2061±199	2414±145**
Leptin (ng/ml)	0.73±0.07	0.65±0.06	1.55±0.10^§^	1.61±0.23^§^	1.69±0.09^§^	0.70±0.08	3.44±0.51^†^ **	3.19±0.51^†^ **	3.43±0.73^†^ **
Food intake (kal/d)	34.1±1.9	38.9±1.9	44.0±1.9	46.6±2.2	73.5±3.1^§^	42.1±2.1	52.2±2.4	61.6±3.1	102.2±3.8**
Insulin (IU/L)	31.9±1.94	45.5±2.06	37.8±4.51	34.3±1.92	36.7±1.27	139.6±9.4**	177.6±19.54**	248.2±15.58^†^ **	194.5±25.47^†^ **
Glucose (mM)	4.3±0.18	6.1±0.47	4.6±0.25	4.8±0. 21	5.0±0.11	9.2±0.31**	9.3±0.29**	9.2±0.23**	8.8±0.26**
HOMA-IR	6.1±0.55	7.1±0.65	7.6±0.86	7.4±0.68	8.1±0.34	55.5±3.46**	74.5±10.50**	101.3±5.31^†^ **	77.7±15.69^†^ **
OGTT-AUC	14.28±0.28	14.89±0.29	ND	ND	ND	14.48±0.26	14.69±0.28	15.48±0.61^§^	15.12±0.48^§^

Blood was drawn after 12 h fasting. Values are means ± SE (n = 6–8 per group).

^§^ *P*<0.05 relative to 0-week under chow diet,

^†^ *P* <0.05 relative to 1-week under HFHS diet;

^**^ *P*<0.01, HFHS diet *versus* chow diet. *TG*, triglyceride; *TC*, total cholesterol; *HDL-c*, high-density lipoprotein associated cholesterol; *LDL-c*, low density lipoprotein associated cholesterol; *FFA*, free fatty acid; *HOMA-IR*, homeostasis model assessment of basal insulin resistance; *OGTT*, area under curve of oral glucose tolerance test; *ND*, Not determined.

Hyperleptinemia occurred at 2-week HFHS dieting (Table 1), one week after the onset of hyperinsulinemia. Food intake increased in both dietary groups as the rats growing, and the HFHS diet-fed rats were profoundly hyperphagic at 8^th^ week despite hyperleptinemia (Table 2). Serum FFA levels were comparable between HFHS and chow dieting during the first 4 weeks and increased by 30% at the end of 8^th^ week HFHS dieting (Table 1), suggesting that the lipolytic function of adipose tissue was not compromised until the late stage of insulin/leptin resistance. The increased fasting FFA concentration at the late stage of HFHS dieting was not associated with an increase in plasma TG. Rather, fasting plasma TG was gradually decreased from 2^nd^ to 8^th^ week of HFHS dieting (Table 1). In a separate experiment where the rats were fed the HFHS diet for up to 12 weeks, fasting plasma TG concentration was further decreased to a level that was lower than that in control animals (chow: 1.62 ± 0.80 mM, HFHS: 0.98 ± 0.16 mM; *P* < 0.01). Assuming that fasting plasma TG concentration reflects secretion of hepatic very low density lipoproteins (VLDL), the decrease in plasma TG probably is indicative of compromised hepatic VLDL production upon prolonged HFHS dieting.

**Table 2:** Liver metabolic parameters and liver enzymes.

Parameters	Chow diet (week)					HFHS diet (week)			
	0	1	2	4	8	1	2	4	8
Liver TG (mg/g)	14.1±0.32	21.7±1.26	20.6±0.9	21.0±0.7	20.2±0.6	44.9±1.6**	55.7±1.7**	56.6±2.0**	58.3±1.2**
Liver TC (mg/g)	6.6±0.36	7.3±0.16	7.5±0.14	7.8±0.26	7.7±0.58	17.0±0.64**	16.8±0.85**	15.6±1.0**	12.7±0.67**
Body weight (g)	180±2.0	205±3.4	280±5.4^§^	317±4.2^§^	369±7.4^§^	211±3.0	283±3.0	331±5.3^†^	434±8.7^†^ **
Liver weight (g)	6.2±0.07	7.6±0.18	7.1±0.06	7. 5±0.09	9.9±0.07^§^	9. 9±0.26**	14.2±0.24^†^ **	15.70±1.2^†^ ^* *^	18.7±0.40^†^ ^**^
Liver/body weight ratio (%)	3.43±0.06	3.82±0.13	3.53±0.03	3.35±0.02	2.69±0.02	4.81±0.16	5.0±0.08**	4.69±0.06**	4.31±0.03**
Liver steatosis score	0.0±0.0	0.1±0.01^§^	0.1±0.01^§^	0.2±0.01^§^	0.2±0.01^§^	0.2±0.01**	0.5±0.03^†^ **	1.2±0.03^†^ **	2.2±0.06^†^ **
Hepatocytes with lipids (%)	0.0±0.0	1.2±0.02^§^	2.2±0.02^§^	2.5±0.04^§^	2.8±0.05^§^	2.6±0.14**	4.6±0.14^†^ **	45.8±0.58^†^ **	85.9±1.00^†^ **
ALT (u/l)	45.6±2.4	36.4±1.7	48.3±5.5	52.3±3.5	51.6±2.8	42.9±1.7	62.5±2.4	52.1±1.5	54.4±2.7
AST (u/l)	174.3±19.4	61.4±1.8^§^	139.7±20.0	133.9±15.8	125.5±12.9	55.0±1.2	112.0±8.1	99.4±7.9	88.8±3.6

Values are means ± SE (n = 6–8 per group).

^§^ *P* < 0.05 relative to 0-week under chow diet,

^†^ *P*<0.05 relative to 1-week under HFHS diet;

^**^ *P*<0.01, HFHS diet *versus* chow diet. *TG*, triglyceride; *TC*, total cholesterol; *ALT*, alanine transaminase; *AST*, aspartate transaminase.

### Changes in hepatic metabolic parameters upon HFHS dieting

**Fig 1:**
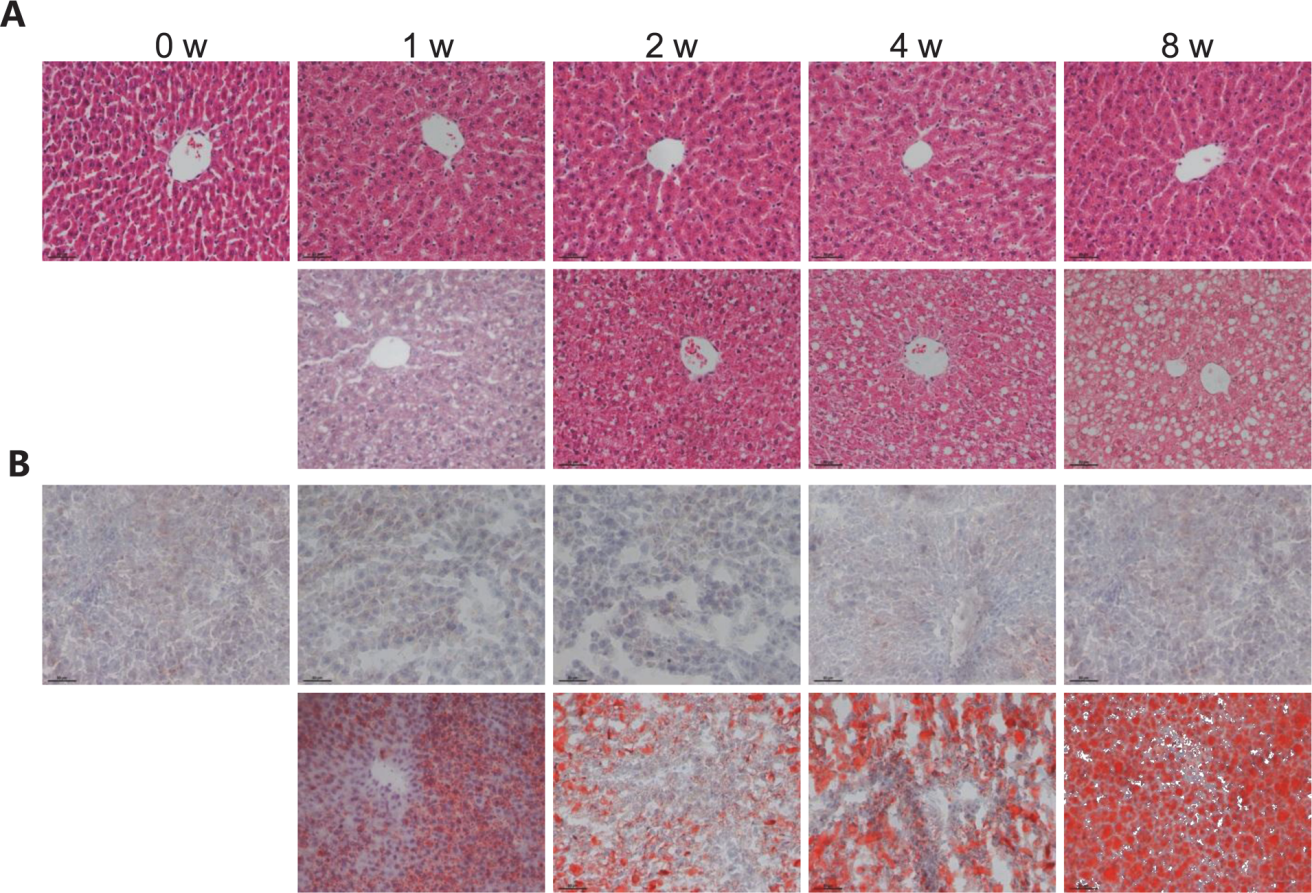
Development of hepatic steatosis upon HFHS dieting. Male Wistar rats (6 weeks old) were placed on chow diet or HFHS diet for 0, 1, 2, 4 and 8 weeks. The livers were excised, processed and stained with hematoxylin-eosin (HE) (A) and Oil Red O (B) (up*per panels*, *chow diet; lower panels*, *HFHS diet*). Images are magnified 200×.

The HFHS dieting also markedly induced hepatic steatosis; elevated liver-associated TG and cholesterol were observed throughout the entire 8-week feeding period (Table 2). Notably, significant increase in hepatic TG and cholesterol was detected as early as ½-week HFHS feeding (TG: 39.1 ± 1.25 *versus* 15.5 ± 0.25 mg/g; TC: 15.71 ± 0.92 *versus* 4.97 ± 0.86 mg/g, *P* < 0.01). Hepatosteatosis in HFHS-fed rats was associated with hepatomegaly, thus the liver-to-body weight ratio was increased immediately 1-week after HFHS dieting, even though there was no significant change in body weight between the two dietary groups until the end of 8^th^ week (Table 2). Although biochemical analysis of hepatic TG and cholesterol showed significant increase upon HFHS dieting, histological analysis did not suggest hepatosteatosis at 1^st^ or 2^nd^ week dieting (Fig. 1A and 1B). The steatosis score, a histological scoring system for NAFLD, of HFHS-fed liver was less than 1, and the extent of hepatocytes with visible lipid accumulation was less than 5% (Table 2). Massive macrovesicles were observed in liver sections at the 4^th^ to 8^th^ week HFHS diet-fed rats (Fig. 1A and 1B), resulting in pathological steatosis scoring (Table 2). Significant inflammatory cells were not found in liver tissues even with 8-week HFHS diet-fed rats (Fig. 1A). Determination of liver enzymes (e.g. ALT and AST) also showed no changes between HFHS and chow diet fed rats (Table 2). These results combined suggested that (i) HFHS dieting induced NAFLD was not detected histologically until 4^th^ to 8^th^ week HFHS dieting, and (ii) there was an absence of overt liver damage or inflammation during the 8-week HFHS dieting. However, accumulation of hepatic TG and cholesterol could be detected biochemically at as early as ½-week HFHS dieting, indicating alterations in lipid metabolism occurred in the liver at least 2 weeks before pathological NAFLD diagnosis.

### Up-regulation of SOCS3 in both hypothalamus and liver upon HFHS dieting

**Fig 2:**
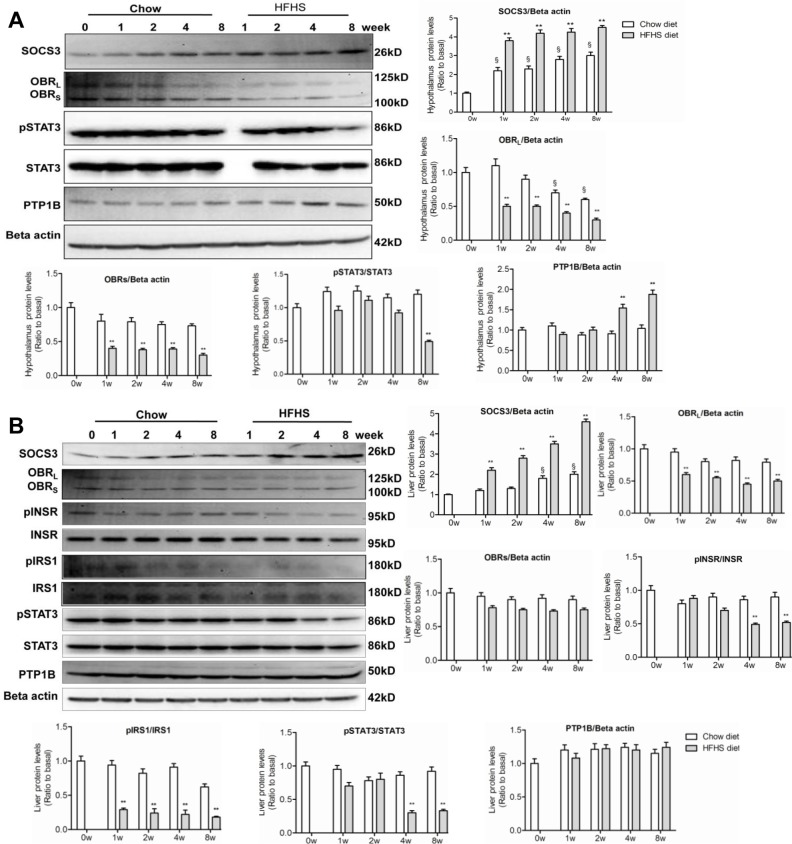
Onset of leptin resistance and lowered insulin sensitivity in hypothalamus and liver upon HFHS dieting. Liver and hypothalamus samples were collected (after 10-hours fasting) at 0, 1, 2, 4 and 8-week feeding with chow or HFHS diet. (A) Western blots of SOCS3, OBR (L and S forms), phospho-STAT3 (pSTAT3), STAT3, and PTP1B in hypothalamus. (B) Western blots of SOCS3, phospho-INSR (pINSR), INSR, phosphor-IRS1 (pIRS1), IRS1, phospho-STAT3 (pSTAT3), STAT3, and PTP1B in the liver. Data (mean ± SE, n = 3) are presented as relative levels compared to that of basal (0 week). ^§^ *P*< 0.05 relative to basal (0 week). ^*^ *P*<0.05, ^**^ *P*<0.01 between HFHS diet versus chow diet conditions.

The abnormalities in lipid/lipoprotein and glucose metabolism upon HFHS dieting were associated with activation of counter-regulatory signaling pathways, including activation of SOCS3. HFHS dieting rapidly induced expression of SOCS3, a negative feedback regulator of leptin and insulin signaling in both central and peripheral target tissues [34]. Western blot analysis revealed that the level of SOCS3 in hypothalamus (Fig. 2A) and the liver (Fig. 2B) was markedly increased as early as 1-week HSHF dieting.

Elevated hypothalamic and hepatic SOCS3 expression was associated with down-regulation of OBR (Fig. 2A and 2B), suggesting an induction of leptin resistance in the respective tissues. However, down-regulation of phosphorylation of STAT3 (the JAK2 effector protein) occurred much later during HFHS dieting as compared to SOCS3 un-regulation. In hypothalamus, decreased STAT3 phosphorylation was observed between 6^th^ (not shown) and 8^th^ week (Fig. 2A) after HSHF dieting. The late onset of STAT3 inactivation shown in the present study was in agreement with previous observations, where attenuated hypothalamic JAK2/STAT3 signaling did not occur until 5^th^ to 6^th^ weeks of high-fat diet [35]. The normal STAT3 activity during the first 4-week HFHS dieting has thus been dubbed as the “early stage” leptin resistance [36].

SOCS3 has also been shown to attenuate hepatic insulin signaling by binding to the insulin-receptor (INSR), interfering IRS phosphorylation [37], or promoting ubiquitin-mediated IRS degradation [38]. Indeed, rapid up-regulation of SOCS3 in the liver upon HFHS dieting (as early as 1-week) was associated with decreased IRS1 phosphorylation (Fig. 2B). STAT3 phosphorylation in hypothalamus decreased at 8-week, and decreased hepatic STAT3 phosphorylation also occurred at 4^th^ week HFHS dieting (Fig. 2B). These results suggest that hypothalamic and hepatic leptin resistance was not manifest immediately upon hyperleptinemia in HFHS-fed rats, and alteration in STAT3 signaling probably exert an effect on lipid or glucose metabolism predominately at late stage of leptin resistance.

It has been shown previously that down-regulation of OBR could also be achieved by PTP1B [20]. Throughout the entire HFHS dieting, there was no change in PTP1B levels in liver as compared to that in chow diet controls (Fig. 2B). Thus, PTP1B may not play a role in the observed down-regulation of hepatic OBR under HFHS conditions. However, in hypothalamus, up-regulation of PTP1B was observed at 4^th^ and 8^th^ week HFHS dieting (Fig. 2A), which might contribute to down-regulation of hypothalamic OBR.

### Changes in signalling pathways involved in gluconeogenesis and lipogenesis upon HFHS dieting

**Fig 3:**
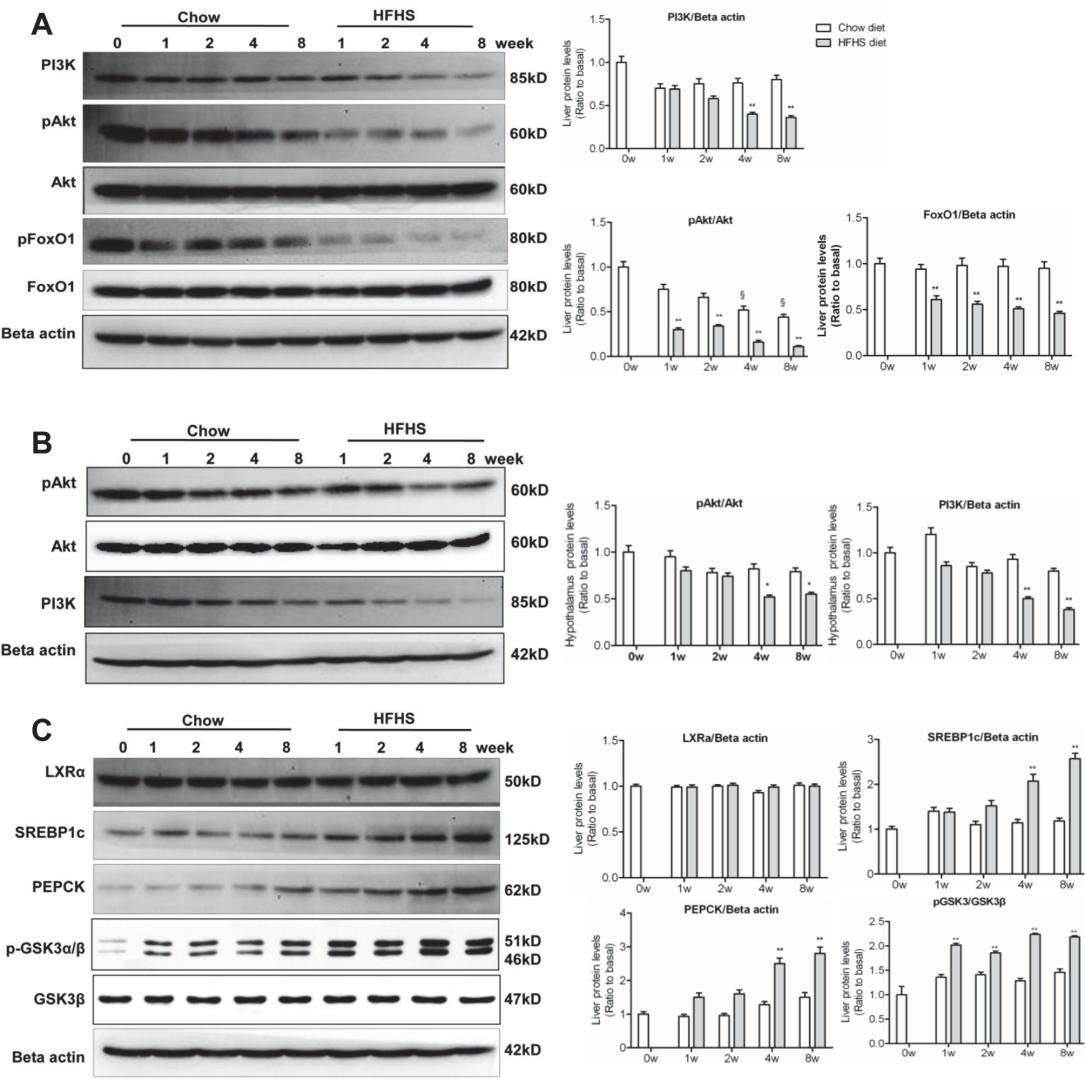
Altered PI3K signalling pathway, lipogenesis, and gluconeogenesis upon HFHS dieting. The experiments were performed the same as described in the legend to Fig. 2. (A) Western blots of PI3Kp85, phospho-Akt (pAkt), Akt, phospho-FoxO1 (pFoxO1) and FoxO1 in the liver. (B) Western blots of phospho-Akt (pAkt), Akt, and PI3Kp85 in hypothalamus. (C) Western blots of LXRα, SREBP1c, PEPCK, phospho-GSK3α/β (pGSK3α/β), and GSK3β in the liver. ^§^ *P*<0.05 relative to basal (0 week). ^*^ *P*<0.05, ^**^ *P*<0.01 between HFHS diet versus chow diet conditions.

The combined impairment in insulin and leptin signaling converged to produce metabolic changes in PI3K, Akt and FoxO1 [20]. In the liver, down-regulation of PI3K, Akt phosphorylation, and FoxO1 was detected as early as 1-week HFHS dieting (Fig. 3A), coinciding with up-regulation of SOCS3 (Fig. 2B). In hypothalamus, down-regulation of PI3K and Akt did not occur until 4^th^ and 8^th^ week HFHS dieting (Fig. 3B), coinciding with down-regulation of hypothalamic STAT3 (Fig. 2A). These results, in agreement with what reported previously [39], suggest that impairment in IRS1/PI3K/Akt pathway occurred earlier than that in STAT3/SOCS3 pathway during insulin/leptin resistance. The present results also suggest that the impairment in IRS1/PI3K/Akt pathway occurred earlier in the liver than in hypothalamus.

The observation of a marked decrease in hepatic FoxO1 upon HFHS dieting (Fig. 3A) was unexpected. Normally upon PI3K/Akt activation, FoxO1 is phosphorylated and excluded from nuclei for degradation [40]. The reason for the observed down-regulation of FoxO1 in the face of decreased PI3K/Akt activation remains to be explained, although leptin has been shown to decrease FoxO1 expression in hypothalamus through PI3K [41]. RT-PCR analysis of gene expression of INSR, IRS1, Akt, and FoxO1 at 1^st^ HFHS dieting showed that there was no difference in their mRNA levels in the livers as compared with chow diet controls (data not shown). These results suggest that the impaired activation of insulin signaling was unlikely the cause of decreased FoxO1 proteins. Rather, the decrease in FoxO1 was likely attributable to accelerated posttranslational degradation.

Up-regulation of PEPCK and SREBP1c was observed in the liver of HFHS-fed rats (Fig. 3C), consistent with the occurrence of lowered hepatic insulin sensitivity and leptin resistance. The precise mechanism or transcription factors involved in PEPCK and SREBP1c expression under HFHS conditions are not entirely clear. However, pronounced up-regulation of PEPCK and SREBP1c in the liver occurred at 4^th^ and 8^th^ week HFHS dieting (Fig. 3C), suggesting that dysregulation of gluconeogenesis and lipogenesis was associated with inactivation of both PI3K/Akt and STAT3 pathways. There was no change in the expression of LXRα between HFHS and chow diet conditions (Fig. 3C), suggesting that LXRα did not contribute to up-regulation of SREBP1c under the current experimental conditions.

Consideration was also given to the possibility that hyperglycemia under HFHS conditions was due to diminished hepatic glycogen synthesis. To test this possibility, we measured the GSK3 phosphorylation status. As shown in Fig. 3C, phospho-GSK3α (Ser^21^) and phospho-GSK3β (Ser^9^) were both markedly elevated in the liver of HFHS diet-fed rats, indicating suppressed activities in glycogen synthesis. Thus, the unchanged OGTT (Table 1), together with elevated PEPCK and GSK3β phosphorylation (Fig. 3C), suggests that HFHS dieting-induced hyperglycemia in the rats is most likely attributable to hepatic glucose production and not glucose utilization.

### Hepatic response to acute insulin/leptin

**Fig 4:**
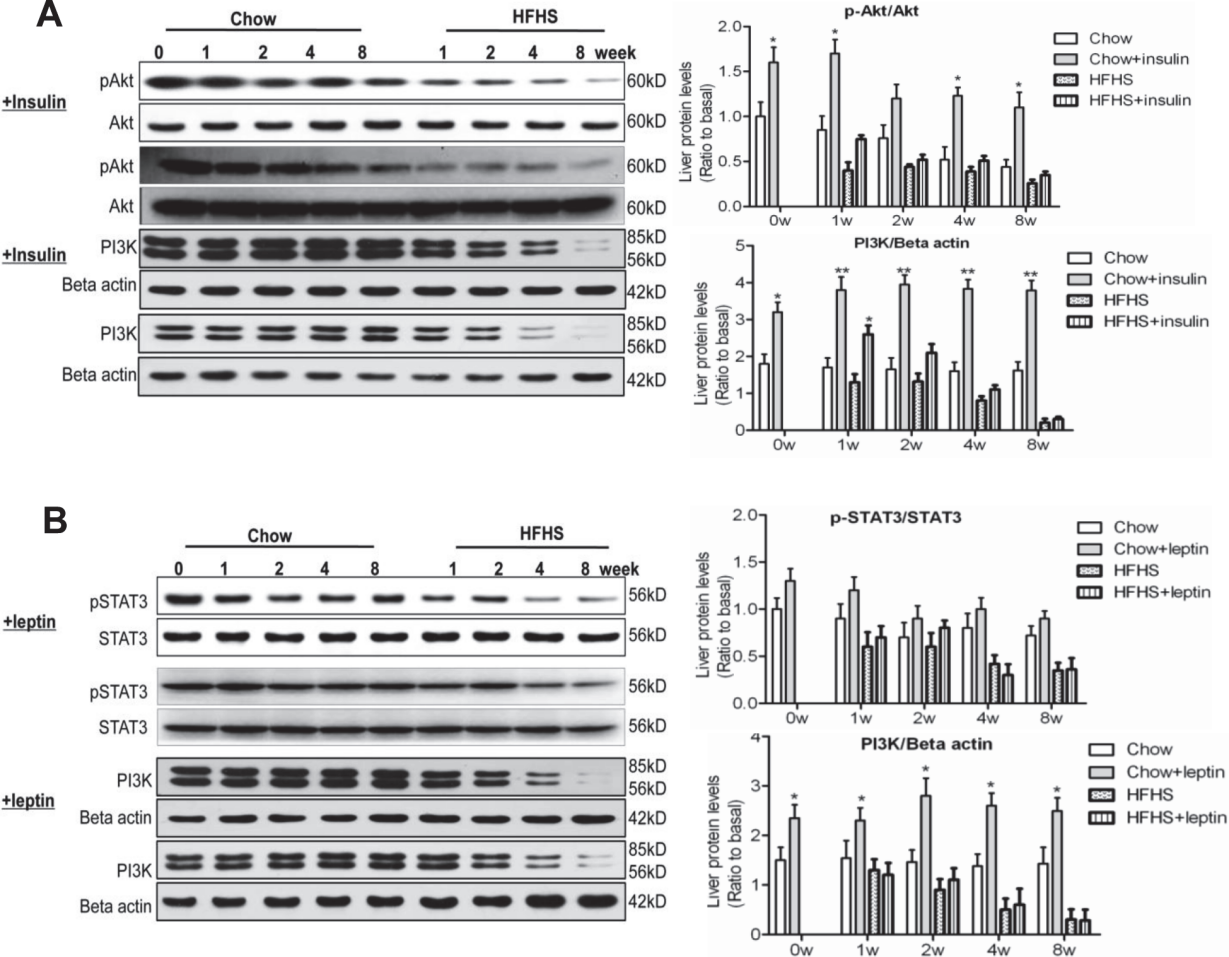
Hepatic response to acute insulin/leptin. At 0, 1, 2, 4 and 8-week feeding with chow or HFHS diet, the animals (12 h fasting) were intraperitoneally injected with insulin (0.75 U/kg body weight) or leptin (0.6 mg/kg body weight), liver samples were collected 30min after the injection. Samples were analyzed along with the non-hormone treated liver samples (A) Western blots of total and phospho-Akt (Thr^308^), PI3Kp85/p55 in the liver 30 min after insulin injection. (B) Western blots of total and phospho-STAT3, PI3K p85/p55 in the liver 30 min after leptin injection. Data (mean ± SE, n = 3) are presented as relative protein levels. ^*^ *P*<0.05, ^**^ *P*<0.01 between hormone treated versus non-hormone treated conditions.

The impaired hepatic insulin/leptin sensitivity was further determined using the rats acutely treated with insulin and leptin, respectively. Hepatic Akt2 phosphorylation under chow diet conditions was markedly increased upon insulin treatment, and such an insulin response was diminished under HFHS diet conditions (Fig. 4A). Likewise, the markedly stimulated PI3Kp85/p55 expression by insulin under chow diet conditions was attenuated after 2-week HFHS feeding (Fig. 4A). These data suggest strongly impaired hepatic insulin signaling by HFHS diet feeding which may contribute to the overall lowered insulin sensitivity in these animals. Acute leptin administration resulted in increased hepatic STAT3 phosphorylation under chow diet conditions. Attenuation of leptin-induced STAT3 phosphorylation under HFHS diet conditions was not obvious at 1^st^ and 2^nd^ week, but became significant at 4^th^ and 8^th^ week of dieting (Fig. 4B). Additionally, HFHS diet feeding markedly diminished leptin-induced PI3K expression as compared with that under chow diet conditions (Fig. 4B). Together, these data confirm that both lowered insulin and leptin sensitivity occurred during the development of hepatic steatosis upon HFHS dieting, and suggest that the impairment in hepatic PI3K/Akt pathway may occur earlier than that in hepatic STAT3/SOCS3 pathway.

## Discussion

The present study utilized a well-studied Wistar male rat model to delineate a temporary relationship between the development of clinical NAFLD, the existence of lowered insulin sensitivity, and the onset of leptin resistance in hypothalamus and the liver, through a continuous feeding with HFHS diet for up to 8 weeks. The acute challenge of insulin or leptin also confirms lowered insulin and leptin sensitivity occurred during the development of hepatic steatosis upon HFHS dieting (Fig. 4), further verifies the existence of impaired signaling transduction. In comparison with those fed with chow diet, the HFHS diet-fed rats manifested metabolic syndromes as early as 1-week dieting. However, the clinical manifestation of NAFLD in these rats, using a histological scoring system developed for humans, did not occur until the end of 4^th^ to 8^th^ week dieting. At the late stage of HFHS dieting, massive hepatomegaly was apparent, which was accompanied with significantly elevated fasting plasma FFA concentrations (e.g. 8-week data in Tables 1 & 2), suggesting a compromised FFA storage in peripheral tissues and increased flux of FFA into the liver. The ability to secrete TG from the liver, presumably in the form of VLDL (as assessed by the fasting plasma TG concentrations) was starting to deteriorate at the late stage of HFHS dieting (Table 1), probably also contributing to the progression of hepatosteatosis as reported previously [42]. Moreover, uncontrolled expression of lipogenesis and gluconeogenesis (as shown by up-regulation of SREBP1c, GSK3β phosphorylation and PEPCK, respectively) in the face of hyperinsulinemia and hyperleptinemia, further exacerbates diabetic dyslipidemia upon prolonged HFHS dieting.

Hepatic gluconeogenesis and lipogenesis are regulated by both the insulin and leptin signaling pathways. Indeed, up-regulation of PEPCK and GSK3β phosphorylation were readily observable in the present study throughout the 8-week HFHS feeding. Hepatic glycogen synthase is regulated by phosphorylation of GSK3β, and GSK3 inhibitors could stimulate hepatic glycogen synthase [43]. The increased GSK3β phosphorylation in our study indicated that reduced hepatic glycogen synthase also contribute to the hyperglycemia. In addition, the increasing fasting glucose levels may also be caused by diminished glucose utilization in peripheral tissues. Impaired insulin signalling could induce glucose transporters (Gluts) diminution, which limits glucose uptake and contributing to the hyperglycemia [44].

Detailed analysis of the main checkpoints of the respective insulin and leptin signaling pathways showed that the occurrence of lowered insulin sensitivity was at least 2 weeks earlier than leptin resistance in both hypothalamus and liver. Specifically, STAT3 activation did not occur until prolonged HFHS dieting (4^th^ week in the liver and 8^th^ week in hypothalamus) even in the presence of hyperleptinemia (which occurred at 2-week of HFHS dieting) and up-regulation of SOCS3 (which occurred as early as 1^st^ week HFHS dieting). Although how does the liver or hypothalamus maintain a relatively normal STAT3 activation during the early stage of insulin/leptin resistance is unclear, an anti-steatogenic effect of hepatic STAT3 has been suggested previously [29,45]. The activation of STAT3 has been shown to play a role in the suppression of PEPCK [46] and SREBP1 expression [47]. The observed elevation in PEPCK and SREBP1c expression at late stage of HFHS dieting (Fig. 3C), coinciding with down-regulation of hepatic STAT3 (Fig. 2B), is consistent with an anti-diabetic, anti-steatogenic role of STAT3. Late suppression of STAT3 phosphorylation in mice fed with a high-fat diet has been reported previously [28,39,48]. Based on these results, it is tempting to speculate that the cellular maintenance of STAT3 activation may represent a hepatic protective mechanism that dampens gluconeogenesis and lipogenesis during early stage of insulin/leptin resistance.

It is well established that STAT3 activation in leptin signaling is attenuated by SOCS3, and decrease in STAT3 activation has been considered as an indicator of leptin resistance. SOCS3 also attenuates the insulin signaling [37,38], representing a cross-talk between the two signaling pathways. The present data showed that the earliest alteration upon HFHS dieting (at 1-week) was up-regulation of SOCS3 in hypothalamus and liver, which coincided with the onset of hyperinsulinemia, hyperglycemia, elevated hepatic TG and cholesterol, down-regulation of OBR, and attenuated insulin receptor activation. However as discussed above, it was noted in the present study that a rapidly up-regulated SOCS3 expression in hypothalamus and liver (at the early stage of HFHS dieting) was not immediately linked to down-regulation of STAT3 activation. Available experimental data indicated that both hyperinsulinemia and SOCS3 contribute to an enhanced lipogenesis through SREBP1c up-regulation [47]. Expression of SOCS3 has been shown to play a central role in hepatic steatosis and insulin resistance in mice [37]. Over-expressing SOCS3 (through adenovirus mediated gene transfer) resulted in up-regulation of PEPCK in mice [37]. On the other hand, STAT3 has the ability to suppresses both SREBP1c and PEPCK, and thus play a critical role in attenuating lipogenesis and gluconeogenesis [49]. These data suggest that SOCS3 and STAT3 regulatory loop was uncoupled initially, leaving a window of opportunity for selective regulation of lipogenesis and gluconeogenesis by the two factors during the early and late stages of leptin/insulin resistance. We hypothesize that the early stage of leptin resistance, manifested by the maintenance of normal STAT3 activity, in the face of up-regulated SOCS3, is probably a compensatory response to the rapidly deteriorated insulin sensitivity under HFHS dieting.

In summary, using HFHS diet-induced NAFLD rat model, we have obtained experimental evidence suggesting that the development of the early stage of NAFLD (without apparent complication of inflammation) is a consequence of uncontrolled hepatic lipogenesis and gluconeogenesis. These metabolic alterations was closely associated with altered insulin/leptin signaling in both hypothalamus and the liver, and the existence of lowered insulin sensitivity and leptin resistance occurred at least 2–3 weeks prior to the manifestation of hepatosteatosis.

## Supporting Information

S1 Table Antibody table. (DOCX)
