# Supplementary material for: Temporal Relationship between Diet-Induced Steatosis and Onset of Insulin/Leptin Resistance in Male Wistar Rats
Source: PLoS One. 2015 Feb 6;10(2):e0117008. doi: 10.1371/journal.pone.0117008 (PMC4319780; doi:10.1371/journal.pone.0117008)
Supplement: S1 Table — (DOCX) [file pone.0117008.s001.docx]

**Table S1**

**Antibody table**

| **Peptide/protein target** | **Name of Antibody** | **Manufacturer, catalog #, and/or name of individual providing the antibody** | **Species raised in; monoclonal or polyclonal** | **Dilution used** |
| --- | --- | --- | --- | --- |
| suppressor of cytokine signaling 3 (SOCS3) | SOCS3 (L210) | Cell Signaling Technology #2932 | Rabbit polyclonal antibody | 1:500 |
| phosphoinositide 3 kinase (PI3K) | PI3K p85 | Cell Signaling Technology #4292 | Rabbit polyclonal antibody | 1:1000 |
| phosphoinositide 3 kinase (PI3K) | PI3K p85/p55 | Cell Signaling Technology #4228 | Rabbit polyclonal antibody | 1:1000 |
| forkhead box protein 1 (FoxO1) | FoxO1 (C29H4) | Cell Signaling Technology #2880 | Rabbit monoclonal antibody | 1:1000 |
| Phosphorylated FoxO1 (Ser256) | Phospho-FoxO1 (Ser256) | Cell Signaling Technology #9461 | Rabbit polyclonal antibody | 1:1000 |
| insulin receptor | Insulin Receptor β (4B8) | Cell Signaling Technology #3025 | Rabbit monoclonal antibody | 1:2000 |
| phosphorylated insulin receptor | phospho-insulin receptor β(Tyr1146) | Cell Signaling Technology #3021 | Rabbit polyclonal antibody | 1:500 |
| insulin receptor substrate 1 | IRS-1 (D23G12) | Cell Signaling Technology #3407 | Rabbit monoclonal antibody | 1:1000 |
| phosphorylated insulin receptor substrate 1 | Phospho-IRS-1 (Ser302) | Cell Signaling Technology #2384 | Rabbit polyclonal antibody | 1:500 |
| protein kinase B (Akt2) | Akt2 (D6G4) | Cell Signaling Technology #3063 | Rabbit monoclonal antibody | 1:2000 |
| phosphorylated AKT | Phospho-Akt (Ser473) (D9E) XP | Cell Signaling Technology #4060 | Rabbit polyclonal antibody | 1:1000 |
| phosphorylated AKT | Phospho-Akt (Thr308) | Cell Signaling Technology #9275 | Rabbit polyclonal antibody | 1:1000 |
| signal transducer and activator of transcription 3 (STAT3) | Stat3 (124H6) Mouse | Cell Signaling Technology #9139 | Mouse monoclonal antibody | 1:2000 |
| phosphorylated STAT3 | Phospho-Stat3 (Tyr705) (D3A7) XP | Cell Signaling Technology #9145 | Rabbit monoclonal antibody | 1:1000 |
| Glycogen synthase kinase-3 (GSK-3) | GSK-3β Antibody (E-11) | Santa Cruz sc-377213 | Mouse monoclonal antibody | 1:200 |
| phosphorylated GSK-3 | Phospho-GSK3α/β (Ser21/9) | Cell Signaling Technology #9331 | Rabbit polyclonal antibody | 1:1000 |
| protein-tyrosine phosphatase 1B (PTP1B) | Anti-PTP1B antibody [EP1837Y] | Abcam ab52650 | Rabbit monoclonal antibody | 1:1000 |
| leptin receptor (OBR) | Anti-Leptin Receptor | Abcam ab5593 | Rabbit polyclonal antibody | 1:1000 |
| liver X recptor alpha (LXR alpha) | Anti-LXR alpha | Abcam ab3585 | Rabbit polyclonal antibody | 1:2000 |
| sterol regulatory element binding protein 1 (SREBP1) | Anti-SREBP1 antibody [2A4] | Abcam ab3259 | Mouse monoclonal antibody | 1:1000 |
| phosphoenolpyruvate carboxykinase (PEPCK) | PEPCK Antibody (H-300) | Santa Cruz sc-32879 | Rabbit polyclonal antibody | 1:50 |
|  |  |  |  |  |
| Beta-Actin | Beta-Actin Antibody (C4) | Santa Cruz sc-47778 | Mouse monoclonal antibody | 1:1000 |
| Anti-rabbit | Anti-rabbit IgG, HRP-linked | Cell Signaling Technology #7074 | goat anti-rabbit IgG | 1:5000 |
| Anti-Mouse | Anti-mouse IgG, HRP-linked | Cell Signaling Technology #7076 | horse anti-mouse IgG | 1:5000 |
